# Psychological Burden and Diabetes Self-Management Behaviour Among Northern Saudi Adults with Type 2 Diabetes: A Cross-Sectional Study

**DOI:** 10.3390/healthcare14172764

**Published:** 2026-09-01

**Authors:** Aseel Awad Alsaidan

**Affiliations:** Department of Family and Community Medicine, College of Medicine, Jouf University, Sakaka 72388, Saudi Arabia; aaalsaidan@ju.edu.sa; Tel.: +966-50-606-5757

**Keywords:** type 2 diabetes mellitus, self-management, self-care, depressive symptoms, primary healthcare, Saudi Arabia

## Abstract

**Background and objectives:** Type 2 diabetes mellitus (T2DM) care depends on sustained self-management behaviour and psychological wellbeing. This study examined diabetes self-management behaviour, depressive symptom burden, and associated demographic factors among adults with T2DM. **Methods:** The present cross-sectional study was conducted among T2DM patients attending 10 primary healthcare centres in the Al-Jouf region, northern Saudi Arabia. Diabetes self-management behaviour was assessed using the Summary of Diabetes Self-Care Activities, and depressive symptoms were assessed using Patient Health Questionnaire-9. Binomial logistic regression identified factors associated with low self-management behaviour and no-to-mild depressive symptom status. **Results:** Of the 350 T2DM patients studied, the mean self-care activity score was 28.38 ± 12.10, and the mean PHQ-9 score was 6.39 ± 3.91. Moderate-to-severe depressive symptoms were present in 87 participants (24.9%). Low self-management behaviour was significantly associated with obesity (Ref: normal body mass index; AOR: 1.47, 95% CI: 1.10–2.06, *p* = 0.041), T2DM duration of 6–10 years (Ref: 0–5 years; AOR: 2.12, 95% CI: 1.18–3.83, *p* = 0.013), other chronic illnesses (Ref: no; AOR: 1.93, 95% CI: 1.18–3.16, *p* = 0.004), and moderate-to-severe depressive symptoms (Ref: none-to-mild; AOR: 1.76, 95% CI: 1.21–3.06, *p* = 0.034). **Conclusions:** Suboptimal diabetes self-management behaviour and depressive symptom burden were observed. Integrating structured self-management education, repeated behavioural counselling, and depressive symptom screening may improve patient-centred diabetes care.

## 1. Introduction

Worldwide, type 2 diabetes mellitus (T2DM) is a significant and increasing public health problem that accounts for a significant contribution of premature morbidity, mortality, disability and burden of the health system. In 2024, an estimated 589 million adults (20–79 years) lived with diabetes, and this figure is expected to reach 853 million by 2050, according to the International Diabetes Federation (IDF) [1,2]. Another study also predicted an increasing prevalence of diabetes, with the Middle East and North Africa regions likely to have a disproportionate burden by 2050 [3]. Despite the transition to the adult Saudi population with its associated increases in sedentary lifestyle, obesity, diet changes, and other cardiometabolic risk factors, T2DM is still a high-priority non-communicable disease in Saudi Arabia [4].

Pharmacological management is but a part of the effective care of T2DM, and the work of maintaining the care is largely dependent on the behaviour of the person with T2DM. Patients should take their medications regularly, eat healthy foods, exercise, monitor their blood sugar levels, manage any risk factors, and follow up with a healthcare professional. Along with the daily management of these tasks, there are also constant behavioural and emotional requirements for patients to maintain glycaemic control and prevent complications [5,6]. Diabetes care guidelines are increasingly person-centred, and behavioural support, self-management education, together with psychosocial care constitute integral aspects of comprehensive diabetes care, not just ancillary services to provide alongside standard diabetes care [6,7].

The psychological aspect of T2DM is clinically significant due to repeated decision making, lifestyle modifications, and the concerns about complications related to living with T2DM as a lifelong condition that needs treatment on a long-term basis. Psychological burden may impact motivation, self-efficacy, illness perception, engagement in treatment, and ability to sustain healthy behaviours. Depressive symptoms are particularly relevant among the psychological challenges faced by T2DM patients as they are common, measurable, clinically relevant, and closely associated with worse diabetes outcomes [8,9,10]. For this research, the psychological burden is defined in a narrow and more specific way to denote depressive symptom burden only, and not the entire psychological morbidity.

Diabetic symptoms and poor diabetes self-management can affect one another in more than one way. Depressive symptoms can lead to low energy, low concentration, low motivation, and low self-confidence, which makes it harder to stick with diet, exercise, medication, and follow-up. On the other hand, the emotional distress and burden of depressive symptoms may be higher from poor glycaemic control, treatment complexity, diabetes-related complications, and perceived difficulty in maintaining diabetes self-care [11,12]. Recent studies indicate that there is a link between depression and self-management problems among T2DM patients, and integration of care and psychological support has been increasingly suggested as a way to help people with T2DM who have psychological needs [13,14].

Additionally, evidence from Saudi Arabia also suggests that the issues of psychological and behavioural factors are not fully discussed with patients suffering from T2DM. Similar relationships among illness perceptions, health-related beliefs, self-care behaviours, and clinical and sociodemographic factors have been reported in previous Saudi studies of adults with T2DM [15,16]. But there is a lack of evidence from Northern Saudi Arabia, especially of psychological burden and the impact on diabetes self-management activities in the general population. The distinct contribution of the current research is the concurrent assessment of depressive symptom burden and diabetes self-management behaviour within routine primary healthcare. This provides a context-specific psychological–behavioural profile of adults with T2DM in Northern Saudi Arabia that may help identify groups requiring additional support in routine care.

Thus, there is a need for additional regional evidence to gain greater insight into the psychological and behavioural profiles of adults with T2DM living in Northern Saudi Arabia. The emotional burden of self-management of diabetes may be more clearly highlighted by framing the burden of depressive symptoms as a specific type of psychological burden and maintaining a clinically measurable yet focused approach to this burden. This evidence can support more integrated outpatient diabetes care by identifying patients who may need additional behavioural counselling and psychological support, or reinforcement of self-management, within routine clinical services. Accordingly, this survey assessed diabetes self-care behaviour and depressive symptom burden and determined their associated factors among adults with T2DM attending primary healthcare centres.

## 2. Materials and Methods

### 2.1. Study Design and Setting

The cross-sectional study was carried out between August 2023 and January 2024 among adults with T2DM who received care at primary healthcare facilities in the Al-Jouf region of northern Saudi Arabia. This study adhered the Strengthening the Reporting of Observational Studies in Epidemiology (STROBE) guidelines (Supplementary Table S1).

### 2.2. Sample Size Determination

The sample size was obtained using the equation (*n* = z^2^pq/e^2^), based on an anticipated prevalence (p) of depressive symptoms among adults with T2DM reported in the regional literature, with a 95% confidence level and 5% allowable error (e). The estimated minimum sample was approximately 345 patients; therefore, 350 patients were finally included in the study.

### 2.3. Recruitment Strategies

This survey enrolled adults (aged ≥18 years) with a confirmed diagnosis of T2DM who attended selected primary healthcare centres in the Al-Jouf region during the study period. A convenience sampling approach was used because recruitment was conducted among eligible patients attending routine diabetes-related visits during the study period, allowing feasible enrolment within the available study setting and timeframe. The 10 primary healthcare centres were also selected conveniently from different areas of the Al-Jouf region to include patients attending PHC settings across different geographic areas. Patients were approached during routine clinic visits and invited to participate until the required sample size was achieved. Overall, 403 eligible individuals were contacted, and 350 submitted complete questionnaires, yielding a response rate of 86.8% and forming the final analytic sample.

### 2.4. Eligibility Criteria

Eligible participants were required to have physician-diagnosed T2DM, understand Arabic and provide informed consent. Patients with type 1 diabetes mellitus, gestational diabetes, severe acute illness, or inability to complete the questionnaire were excluded.

### 2.5. Ethical Approval and Participant Protection

This study received ethical approval from Ministry of Health Research Ethics Committee, Al-Qurayyat, Al-Jouf Region, Saudi Arabia [Reg no: H-13-S-071, dated 25 May 2023]. Administrative permission was obtained from the relevant primary healthcare authorities. Before enrolment, participants received the study aims, voluntary involvement, anonymous data, and right to discontinue participation at any time. Informed consent was obtained before participation.

### 2.6. Data Collection Procedure

The questionnaire included sections on sociodemographic characteristics, clinical characteristics, diabetes self-management behaviour, and depressive symptoms. Sociodemographic variables included age, gender, educational status, occupation, monthly income, marital status, and smoking status. Clinical variables included body mass index (BMI) category, duration of T2DM, presence of other chronic illnesses, and type of diabetes medication used (oral/insulin/both).

Diabetes self-care behaviour was determined by the Arabic version of the Summary of Diabetes Self-Care Activities (SDSCA) tool adapted from previously published studies among Arabic-speaking adults with T2DM [12,17,18]. The SDSCA was originally developed to assess diabetes self-care activities [19] and has been psychometrically evaluated in Arabic/Saudi settings [Cronbach’s alpha (α) = 0.76] [18]. In the present sample, the SDSCA indicated good internal consistency (α = 0.83). The SDSCA assesses self-care activities undertaken over the preceding seven days in major areas of diabetes management, including dietary practices, physical activity, blood glucose monitoring, foot care, and medication-related practices. In the present study, 15 items were used, and each item was scored according to the number of days the activity was performed during the previous week, from 0 to 7 days. Negatively worded items were reverse scored where applicable. The total score ranged from 0 to 105, with higher scores indicating better diabetes’ self-management behaviour. The distribution of the total self-management behaviour score was assessed and found to be non-normal (Applied test: Shapiro–Wilk’s). As no established clinical cut-off is available for the SDSCA total score, the sample median was used as an operational cut-off to distinguish comparatively lower and higher levels of self-management within the study population. This categorisation was selected for the primary regression analysis to facilitate identification and interpretation of factors associated with lower versus higher self-management behaviour. Similar median-based categorisation of SDSCA/self-care scores has been used in previous studies among adults with T2DM [20,21].

Depressive symptoms were measured with the Arabic version of the Patient Health Questionnaire-9 (PHQ-9), that was validated in a Saudi sample [22]. The PHQ-9 is a brief self-reported screening tool for depressive symptom severity and comprises nine items corresponding to depressive symptoms experienced over the preceding two weeks. Each item is scored from 0 (not at all) to 3 (nearly every day), giving a total score ranging from 0 to 27. Standard PHQ-9 depressive symptoms severity categories were used: none/minimal (0–4), mild (5–9), moderate (10–14), moderate to severe (15–19), and severe (20–27). For regression analysis, depressive symptom burden was dichotomised into none-to-mild (PHQ-9 score 0–9) and moderate-to-severe (PHQ-9 score 10–27), including moderate, moderately severe, and severe categories. This grouping was used because a PHQ-9 score of 10 or above is a commonly used threshold for clinically relevant moderate-to-severe depressive symptoms in the previous literature [23,24]. In the present sample, the PHQ-9 showed acceptable internal consistency (α = 0.79). Data were collected in the primary healthcare centres by the research team or trained data collectors. Participants completed the questionnaire in Arabic. Assistance was provided when needed to clarify the meaning of questions, without influencing participants’ responses.

### 2.7. Data Analysis

Statistical analyses were performed using IBM SPSS Statistics, version 21.0 (IBM Corp., Armonk, NY, USA). Categorical data are summarised as counts and proportions. Continuous data are expressed using mean ± standard deviation. The distribution of continuous outcome scores was assessed before categorisation. Diabetes self-management behaviour item scores and total scores were additionally summarised by median and interquartile range (IQR). Binomial logistic regression with the enter approach was applied to examine factors associated with low diabetes self-management behaviour and no-to-mild depressive symptom status. Separate regression models were used for depressive symptom status and self-management behaviour status. Covariates comprised age group, gender, educational status, occupation, monthly income, marital status, smoking status, BMI category, duration of T2DM, presence of other chronic illness, and type of diabetes medication. Multicollinearity was not evident, as tolerance and VIF values remained within acceptable limits. The Hosmer–Lemeshow test indicated adequate model fit, and the Nagelkerke R^2^ showed acceptable explanatory performance. Examination of standardised residuals and Cook’s distance did not identify influential observations that substantially affected the models. Findings are expressed as adjusted odds ratios (AORs) and corresponding 95% confidence intervals (CIs). Statistical significance was set as *p* < 0.05. Only datasheets with complete responses were retained in the final analysis; therefore, there were no item-level missing data in the analysed dataset, and no imputation was required.

## 3. Results

Among the study participants, the highest proportion were aged 50–60 years (146, 41.7%), male (195, 55.7%), educated up to high school level (198, 56.6%), employed in the private sector (135, 38.6%), and had a family monthly income of more than 7000 SAR (125, 35.7%). Most participants were married (312, 89.1%), non-smokers (295, 84.3%), overweight (129, 36.9%), had T2DM for 6–10 years (137, 39.1%), had no other chronic illnesses (229, 65.4%), and were treated with oral hypoglycaemic agents (192, 54.9%) (Table 1).

Table 2 depicts the diabetes self-care activity items among the study participants; the highest mean score was observed for spreading carbohydrate intake appropriately across the day (mean: 2.69, SD: 1.99; median: 2, IQR: 1–4), followed by drying carefully between the toes (mean: 2.67, SD: 2.27; median: 2, IQR: 1–4). The lowest mean score was observed for checking footwear before wearing (mean: 0.82, SD: 0.65; median: 1, IQR: 0–1).

Regarding depressive symptom categories, the highest proportion of participants had no depressive symptoms (191, 54.6%), followed by mild depressive symptoms (72, 20.6%) (Table 3).

The mean diabetes self-care activity score was 28.38 ± 12.10, with a median score of 27 and an IQR of 20–36. The mean PHQ-9 score was 6.39 ± 3.91, with a median score of 4 and an IQR of 2–9 (Table 4).

The adjusted odds ratios (AORs) in this model represent the odds of having low diabetes self-management behaviour. Up to high school education was significantly associated with lower odds of low self-management behaviour (Ref: no formal education; AOR: 0.73, 95% CI: 0.58–0.90, *p* = 0.021). Obesity was significantly associated with higher odds of low self-management behaviour (Ref: normal BMI; AOR: 1.47, 95% CI: 1.10–2.06, *p* = 0.041). T2DM duration of 6–10 years (Ref: 0–5 years; AOR: 2.12, 95% CI: 1.18–3.83, *p* = 0.013) and more than 10 years (AOR: 1.87, 95% CI: 1.17–3.27, *p* = 0.029) were significantly associated with higher odds of low self-management behaviour. The presence of other chronic illnesses was significantly associated with higher odds of low self-management behaviour (Ref: no other chronic illnesses; AOR: 1.93, 95% CI: 1.18–3.16, *p* = 0.004). Moderate-to-severe depressive symptoms were also significantly associated with higher odds of low self-management behaviour (Ref: none-to-mild depressive symptoms; AOR: 1.76, 95% CI: 1.21–3.06, *p* = 0.034) (Table 5).

The AORs in this model represent the odds of having no-to-mild depressive symptoms. Age 50–60 years was significantly associated with higher odds of no-to-mild depressive symptoms (Ref: <50 years; AOR: 2.74, 95% CI: 1.14–4.61, *p* = 0.024). Female gender was significantly associated with lower odds of no-to-mild depressive symptoms (Ref: male; AOR: 0.47, 95% CI: 0.24–0.71, *p* = 0.025). Obesity was significantly associated with lower odds of no-to-mild depressive symptoms (Ref: normal BMI; AOR: 0.54, 95% CI: 0.39–0.76, *p* = 0.009). The presence of other chronic illnesses was significantly associated with lower odds of no-to-mild depressive symptoms (Ref: no other chronic illnesses; AOR: 0.61, 95% CI: 0.37–0.88, *p* = 0.031). Both types of medicine use were significantly associated with lower odds of no-to-mild depressive symptoms (Ref: oral hypoglycaemic agents; AOR: 0.55, 95% CI: 0.37–0.68, *p* = 0.017). High self-management behaviour was significantly associated with higher odds of no-to-mild depressive symptoms (Ref: low self-management behaviour; AOR: 1.31, 95% CI: 1.09–1.82, *p* = 0.037) (Table 6).

## 4. Discussion

The T2DM care is not limited to glycaemic control alone but also depends on patients’ ability to sustain daily self-management behaviours while coping with the psychological burden of a chronic disease. Evidence from Northern Saudi Arabia remains limited regarding how diabetes self-management behaviour and depressive symptom burden coexist among adults with T2DM in primary healthcare settings. Therefore, this study adds regional evidence on self-care activity patterns, depressive symptom burden, and their associated demographic and clinical factors.

The present study revealed suboptimal diabetes self-management behaviour with a greater rate of suboptimal behaviour in more structured and preventive diabetes self-care domains. A recent study indicated a positive association between health literacy and diabetes knowledge, diabetes self-care, and glycaemic control [25]. Thus, the lower self-management pattern found in the present survey may, in part, be attributed to variations in health literacy, education, counselling exposure, and diabetes advice to convert into repeated daily self-management. In a multinational study on barriers and enablers for diabetes self-management, emotional burden, social support, access to services, patient–provider communication, and competing daily burdens were identified as factors influencing diabetes self-management [26]. This is consistent with the current study, which indicated that self-care behaviour was low despite having the ability to access routine primary healthcare services. Variations across studies may be attributable to the type of healthcare setting, cultural context, methods used to measure self-care, and the approach of measuring the total self-care behaviour, domain-specific behaviour, or adherence to selected recommendations.

Physical activity and foot care differ from taking medication or receiving general dietary advice in that they require motivation, risk-taking, skill, and reinforcement over time. Modern research on diabetes care has focused on the importance of offering practical, patient-centred, self-management support that goes beyond general instructions to include addressing barriers to behaviour change, problem-solving ability, and the individual context in which people are living [27,28]. Therefore, the current results suggest that primary healthcare counselling could extend beyond general lifestyle information to reinforce specific exercise, blood sugar, and foot care behaviours.

This current study demonstrated the relevance of depressive symptom burden in adults with T2DM in the primary healthcare setting. This result is typically less than that in populations with diabetes in more complex clinical or intervention settings, where psychological distress, diabetes distress, and depressive symptoms can be more common [29,30]. The lower burden in the present study may be due to the primary healthcare setting and the use of the PHQ-9 categorisation as opposed to diabetes-distress measures. This is clinically relevant as depressive symptoms, and diabetes distress have been associated with decreased motivation, self-efficacy, treatment adherence, and self-care maintenance [27,29]. Thus, although depression symptom burden is less than in some previous reports, routine screening for depressive symptoms in primary-healthcare diabetes care may be considered as part of comprehensive diabetes care.

In the current study, other chronic illnesses, depressive symptoms symptom burden, duration of T2DM, and educational status were found to be associated with low levels of diabetes self-management behaviour. The results are in line with recent studies indicating that patient education, access to structured counselling sessions, physical activity capacity, multimorbidity, and the burdens and practicalities of maintaining long-term care routines are factors that influence diabetes self-management [31,32,33]. The link with the level of education could be related to a different comprehension of dietary recommendations, glucose-monitoring guidelines, drug timing, or foot-care prevention messages. This is in line with recent evidence on self-management that patients need to receive practical education, repeated education, and contextualised education, not just a single piece of general advice [31,34]. The relationships with obesity and longer diabetes duration may be due to treatment fatigue, decreased physical activity, difficulty maintaining a diet, and increasing complexity of treatment. The latter concords with recent physical-activity recommendations for T2DM that recommend tailoring behaviour change to functional capacity, weight-related barriers, and comorbidity profile [32,35].

There is recent evidence that self-management can become more challenging when patients have multiple chronic conditions to manage [33,34]. The link with depressive symptom burden also may reflect an association with difficulties in energy, planning, and adherence to diabetes self-care.

In the depressive symptom-status model, age, gender, obesity, other chronic illnesses, insulin use, and self-management status were found to be associated with depressive symptom status. The gender-related finding is also consistent with more recent international evidence indicating that women are more likely to suffer from depressive symptoms and anxiety disorders, which could be attributed to biological, social, caregiving and stress factors [36]. The link with obesity is also clinically plausible, as obesity may exacerbate functional limitation, body-image issues, sleep dysfunction, and the ability to reach lifestyle goals, findings that were supported by recent global evidence, which shows that obesity continues to be a significant metabolic and public health burden [37].

This is consistent with recent evidence on multimorbidity that indicates that those who have multiple chronic conditions have a higher complexity of care and a heavier burden of self-management [33,38]. Insulin use could simply be a marker for the presence of more advanced disease and increased treatment complexity, along with an increased risk of hypoglycaemia and/or greater need for increased monitoring, rather than a direct cause of depressive symptoms [35]. Finally, the link between high self-management behaviour and no-to-mild depressive symptoms gives reason to believe there is a close behavioural–psychological relationship. Higher self-management and lower depressive symptom burden may coexist with better perceived control and adherence to diet, physical activity, medications, glucose monitoring, and foot-care practices. But the cross-sectional nature of the present study does not allow for causal interpretation.

This study has several strengths, including addressing the regional evidence gap, relevant routine diabetes care, and identification of demographic and clinical factors associated with low diabetes self-management behaviour and depressive symptom status using regression analysis. However, readers of this paper should consider the following limitations. Firstly, the cross-sectional design prevents causal inference regarding the relationship between depressive symptoms and diabetes self-management behaviour. Secondly, the use of convenience sampling from selected primary healthcare centres may limit the generalisability of the findings to all adults with T2DM in Saudi Arabia and other Middle Eastern countries. Next, bias due to self-reported responses, such as recall, and social desirability bias cannot be ignored. In addition, this study did not include several clinical variables, such as HbA1C and diabetes complications. Furthermore, the median-based dichotomisation of the SDSCA score may have resulted in loss of information and statistical power. Moreover, the PHQ-9 questionnaire is not a clinical diagnosis confirmation tool. Therefore, future studies should therefore use longitudinal, multicentre designs with probability-based sampling across broader geographic areas and combine self-reported measures with objective clinical indicators and structured psychological assessment. Finally, residual confounding from unmeasured factors, including diabetes distress, health literacy, social support, cognitive status, healthcare access, and broader socioeconomic context, cannot be excluded.

## 5. Conclusions

The present study indicated that the quality of diabetes self-care in adults with T2DM visiting PHC centres in Northern Saudi Arabia was not optimal, and depressive symptom burden was present in a significant number of the patients. Educational status, obesity, the number of years since the onset of T2DM, other chronic conditions, and moderate-to-severe depressive symptoms were associated with a low level of self-management behaviour. Demographic and clinical characteristics, such as gender, obesity, comorbidity, insulin use, and self-management behaviour, were also associated with depressive symptom status. The results highlight the potential relevance of structured self-management education, repeated behavioural counselling, and screening for depressive symptoms in primary healthcare services. Psychological support could be combined with counselling for diabetes self-management to recognise at-risk patients and support patient-centred care for diabetes in this region. Finally, future longitudinal and multicentre studies are suggested that include clinical indicators and structured psychological evaluation.

## Figures and Tables

**Table 1:** Baseline profile of the T2DM patients (*n* = 350).

Variable	Frequency	Proportion
Age groups (years)		
<50	71	20.3
50 to 60	146	41.7
>60	133	38.0
Gender		
Male	195	55.7
Female	155	44.3
Education status		
No formal education	48	13.7
Up to high school	198	56.6
University and above	104	29.7
Occupation		
Government sector	112	32.0
Private sector	135	38.6
Retired/Unemployed	103	29.4
Family monthly income *		
<5000 SAR	110	31.4
5000 to 7000 SAR	115	32.9
>7000 SAR	125	35.7
Marital status		
Single	38	10.9
Married	312	89.1
Smoking status		
No	295	84.3
Yes	55	15.7
Body mass index (BMI)		
Normal	108	30.9
Overweight	129	36.9
Obese	113	32.3
Duration of T2DM (years)		
0–5	113	32.3
6–10	137	39.1
>10	100	28.6
Presence of other chronic illnesses		
No	229	65.4
Yes	121	34.6
Treatment		
Oral hypoglycaemic agents (OHAs)	192	54.9
Insulin	90	25.7
Both	68	19.4

* 1 USD = 3.75 SAR.

**Table 2:** Item-wise diabetes self-care activity scores grouped by SDSCA domain.

Main Category	Sub-Questions	Mean	Standard Deviation (SD)	Median	IQR
Diet-related self-care	Followed a generally healthy eating pattern	1.68	1.90	1	0–3
	Kept to the diabetes-specific dietary advice given	1.53	1.83	1	0–2
	Included fruits and vegetables in the diet	2.19	1.71	2	1–3
	Reduced intake of foods high in fat	2.37	1.88	2	1–3
	Spread carbohydrate intake appropriately across the day	2.69	1.99	2	1–4
Physical activity	Performed physical activity for at least 30 min	1.72	1.75	1	1–2
	Took part in a planned exercise session	1.16	1.51	1	1–2
Blood glucose monitoring	Checked blood glucose level	2.02	1.72	2	1–3
	Monitored blood glucose as advised	1.46	1.66	1	0–1
Foot care	Examined the feet	0.99	0.93	1	0–1
	Checked footwear before wearing	0.82	0.65	1	0–1
	Washed the feet	2.66	2.34	2	1–4
	Avoided soaking the feet	1.76	1.55	1	1–3
	Dried carefully between the toes	2.67	2.27	2	1–4
Medication use	Took diabetes medicines as instructed	2.66	2.15	2	1–4

Note. Scores represent the number of days the activity was performed during the previous seven days.

**Table 3:** Depressive symptoms categories according to the PHQ-9 (*n* = 350).

PHQ-9	Frequency	Proportion
None	191	54.6
Mild	72	20.6
Moderate	31	8.9
Moderate severe	40	11.4
Severe	16	4.6
Total	350	100

**Table 4:** Descriptive data of SDSCA and PHQ-9.

Domain	Mean	SD	Median	IQR
Self-care activity behaviours	28.38	12.10	27	20–36
PHQ-9	6.39	3.91	4	2–9

**Table 5:** Factors associated with low diabetes self-management behaviour among T2DM patients (*n* = 350).

Characteristics	Total	Self-Management Behaviours		Binomial Logistic Regression	
		Low (*n* = 166)	High (*n* = 184)	Adjusted OR (AOR) (95% CI)	*p*-Value
Age groups					
<50	71	32	39	Ref	
50 to 60	146	70	76	1.14 (0.53–2.44)	0.731
>60	133	64	69	1.06 (0.61–1.87)	0.832
Gender					
Male	195	87	108	Ref	
Female	155	79	76	1.11 (0.64–1.92)	0.708
Education status					
No formal education	48	34	14	Ref	
Up to high school	198	93	105	0.73 (0.58–0.90)	0.021
University and above	104	39	65	0.56 (0.30–1.05)	0.070
Occupation					
Government sector	112	53	59	Ref	
Private sector	135	69	66	1.65 (0.65–4.32)	0.233
Retired/Unemployed	103	44	59	1.18 (0.88–2.53)	0.397
Family monthly income					
<5000 SAR	110	63	47	Ref	
5000 to 7000 SAR	115	52	63	0.71 (0.35–1.42)	0.331
>7000 SAR	125	51	74	1.01 (0.55–1.85)	0.983
Marital status					
Single	38	16	22	Ref	
Married	312	150	162	1.50 (0.71–3.23)	0.294
Smoking status					
No	295	138	157	Ref	
Yes	55	28	27	1.56 (0.79–3.06)	0.197
BMI					
Normal	108	52	56	Ref	
Overweight	129	59	70	1.07 (0.60–1.91)	0.819
Obese	113	55	58	1.47 (1.10–2.06)	0.041
Duration of T2DM (years)					
0–5	113	47	66	Ref	
6–10	137	61	76	2.12 (1.18–3.83)	0.013
>10	100	58	42	1.86 (1.17–3.27)	0.029
Presence of other chronic illnesses					
No	229	99	130	Ref	
Yes	121	67	54	1.93 (1.18–3.16)	0.004
Treatment					
OHA	192	90	102	Ref	
Insulin	90	42	48	0.98 (0.57–1.76)	0.892
Both	68	34	34	0.89 (0.45–1.78)	0.741
PHQ-9 categories					
No to Mild	263	122	141	Ref	
Other categories	87	44	43	1.76 (1.21–3.06)	0.034

**Table 6:** Factors associated with no-to-mild depressive symptom status among T2DM patients (*n* = 350).

Characteristics	Total	Depressive Symptoms Categories (PHQ-9)		Binomial Logistic Regression	
		No to Mild (*n*= 263)	Other Categories (*n* = 87)	AOR (95% CI)	*p*-Value
Age groups					
<50	71	46	25	Ref	
50 to 60	146	107	39	2.74 (1.14–4.61)	0.024
>60	133	110	23	1.78 (0.89–3.58)	0.105
Gender					
Male	195	156	39	Ref	
Female	155	107	48	0.47 (0.24–0.71)	0.025
Education status					
No formal education	48	34	14	Ref	
Up to high school	198	154	44	1.19 (0.41–3.51)	0.749
University and above	104	75	29	1.25 (0.59–2.64)	0.558
Occupation					
Government sector	112	74	38	Ref	
Private sector	135	104	31	1.85 (0.78–4.37)	0.158
Retired/Unemployed	103	85	18	0.94 (0.43–2.07)	0.867
Family monthly income					
<5000 SAR	110	82	28	Ref	
5000 to 7000 SAR	115	86	29	1.36 (0.58–3.18)	0.481
>7000 SAR	125	95	30	1.07 (0.51–2.23)	0.868
Marital status					
Single	38	29	09	Ref	
Married	312	234	78	0.84 (0.34–2.11)	0.713
Smoking status					
No	295	218	77	Ref	
Yes	55	45	10	1.54 (0.63–3.80)	0.347
BMI					
Normal	108	75	33	Ref	
Overweight	129	100	29	1.24 (0.62–2.47)	0.541
Obese	113	88	25	0.54 (0.39–0.76)	0.009
Duration of T2DM (years)					
0–5	113	85	28	Ref	
6–10	137	99	38	1.220 (0.59–2.53)	0.541
>10	100	79	21	1.731 (0.87–3.43)	0.165
Presence of other chronic illnesses					
No	229	181	48	Ref	
Yes	121	82	39	0.61 (0.37–0.88)	0.031
Treatment					
OHA	192	165	27	Ref	
Insulin	90	60	30	2.02 (0.97–4.25)	0.062
Both	68	38	30	0.55 (0.37–0.68)	0.017
Self-management					
Low	166	122	44	Ref	
High	184	141	43	1.31 (1.09–1.82)	0.037

## Data Availability

The original contributions presented in this study are included in the article/Supplementary Materials. Further inquiries can be directed to the corresponding author.

## Supplementary Materials

The following supporting information can be downloaded at: https://www.mdpi.com/article/10.3390/healthcare14172764/s1, Table S1: STROBE Statement.
